# Origin of Hofmeister Effects for Complex Systems

**DOI:** 10.1371/journal.pone.0128602

**Published:** 2015-07-22

**Authors:** Rui Tian, Gang Yang, Ying Tang, Xinmin Liu, Rui Li, Hualing Zhu, Hang Li

**Affiliations:** College of Resources and Environment, Chongqing Key Laboratory of Soil Multi-scale Interfacial Process, Southwest University, Beibei, Chongqing, P.R. China; University of Akron, UNITED STATES

## Abstract

Hofmeister effects have been recognized as important as Mendel’s work was to genetics while remain largely controversial, especially for the mechanistic aspects. Here we demonstrated that complex colloids in electrolyte solutions show resembling aggregation kinetics as model colloid, and then quantitatively evaluated the resulting Hofmeister effects. Mechanism for the aggregation of complex colloids has been proposed that is closely associated with the charges of their constituents; despite that, electrostatic interactions play a minor role while polarization effect is evidenced to be the driving force for the aggregation processes. Polarization effect is further ascribed to arouse the resulting Hofmeister effects, which is supported by the fine correlation of activation energies vs. polarizability data of different alkali ions and the calculations of dipole moments for minerals with different charges and adsorbed alkali ions. Because of neglecting polarization effect, the prevailing DLVO theory is not sufficient to describe Hofmeister effects that are ubiquitous in nature. We speculate that polarization effect should also be responsible for Hofmeister effects of other charged systems such as proteins and membranes.

## Introduction

Proteins, membranes, clays and colloids carry a plethora of surface charges that result in strong electric fields and particular adsorption behaviors [1–3]. Interactions between colloidal particles can lead to aggregation, and electrolytes dissolved in aqueous solutions have an eminent influence on such aggregation processes, which are known to be Hofmeister effects (specific ion effects) [4–7]. Hofmeister effects were first observed in the 1880s [8] and currently are experiencing a renaissance. People have gradually recognized that Hofmeister effects can be as important in the scheme of things as Mendel’s work was to genetics [9]. Although it is evident that Hofmeister effects are associated with surface charges, mechanistic understanding remains a subject of burgeoning debates [1, 6, 10–13].

A number of previous studies focused on the aggregation of mono-dispersive synthetic colloids (small and spherical particles) [14–17]. Recently, we have used the dynamic light scattering technique to quantitatively characterize the Hofmeister effects for the aggregation of (poly-dispersive) montmorillonite particles in electrolyte solutions [18]. Unfortunately, the prevailing Derjaguin, Landau, Verwey and Overbeek (DLVO) theory is unable to give a reasonable interpretation for the experimentally observed Hofmeister effects [7, 19–24]. As is known to us, natural soil colloids (NSCs) can be composed of a variety of different minerals that support plant life, store water and inhabit environment for living organisms [25, 26]. Many important processes occurring in NSCs, such as adsorption, dispersion, aggregation, precipitation, transport and migration, are closely associated with metal ions [27–29]. For instance, the aggregation of NSCs is a focusing topic in chemical, environmental, soil and colloidal sciences, while to the best of our knowledge, most studies have been performed on the relatively simple and one-component model systems (e.g., montmorillonite and kaolinite) [26, 30, 31]. Because of the potential “huge gaps”, misleading results can be caused when extending the conclusions of model systems to real systems. Here we presented the aggregation kinetics of NSCs in alkali ion solutions, a representative of real and complex systems. Then Hofmeister effects implicated therein were demonstrated, both qualitatively (by giving Hofmeister series) and quantitatively.

As demonstrated in the previous work [18], ionic size [32], hydration [32, 33], dispersion force [34–36] and surface charge [6, 11] failed to interpret the Hofmeister effects arising from the aggregation of montmorillonite. Polarization effect was then proposed while direct evidence is obviously lacking. Hofmeister effects of electrolyte cations and anions have been the concerns of different studies [18, 37–43], and it has been confirmed that whether for NSCs or for montmorillonite, the aggregation processes are dominated by electrolyte cations rather than anions [18, 42]. Accordingly, in this work we will focus on electrolyte cations, and mechanism for the aggregation of NSCs was proposed by use of first-principles density functional calculations. To our surprise, the aggregation mechanism has no close relation with electrostatic interactions, albeit the surface charges have been acknowledged to be the key factor for colloidal formation and stability. On such basis, the origin of Hofmeister effects implicated therein was explored and unambiguously ascribed to polarization effect. We speculate polarization effect should be responsible for Hofmeister effects of other charged systems that are ubiquitous in nature, such as proteins and membranes.

## Materials and Methods

### Dynamic light scattering (DLS) experiments

Presently, two NSCs were considered, as yellow earth soil colloid (NSC1) and purple soil colloid (NSC2). NSCs were prepared as described in the supplementary information or elsewhere [18]. Then the BI-200SM multi-angle DLS instrument (Brookhaven Instruments Corporation, New York, USA) with the BI-9000AT auto-correlator was used to *in situ* monitor the hydrodynamic diameter growths of NSCs. The laser device was polarized vertically with a wavelength of 532 nm. NSCs were sonicated for 2 min, and the various alkali ion solutions were respectively added. The alkali ion concentrations are 20 ∼ 150 mmol/L for LiNO_3_, 10 ∼ 150 mmol/L for NaNO_3_, 5 ∼ 150 mmol/L for KNO_3_, 5 ∼ 150 mmol/L for RbNO_3_ and 5 ∼ 150 mmol/L for CsNO_3_, respectively. After thorough mixing, the hydrodynamic diameters of NSCs were recorded every 30 sec at a scattering angle of 90° (298 ± 1 K).

### Computational methodologies

**Fig 1:**
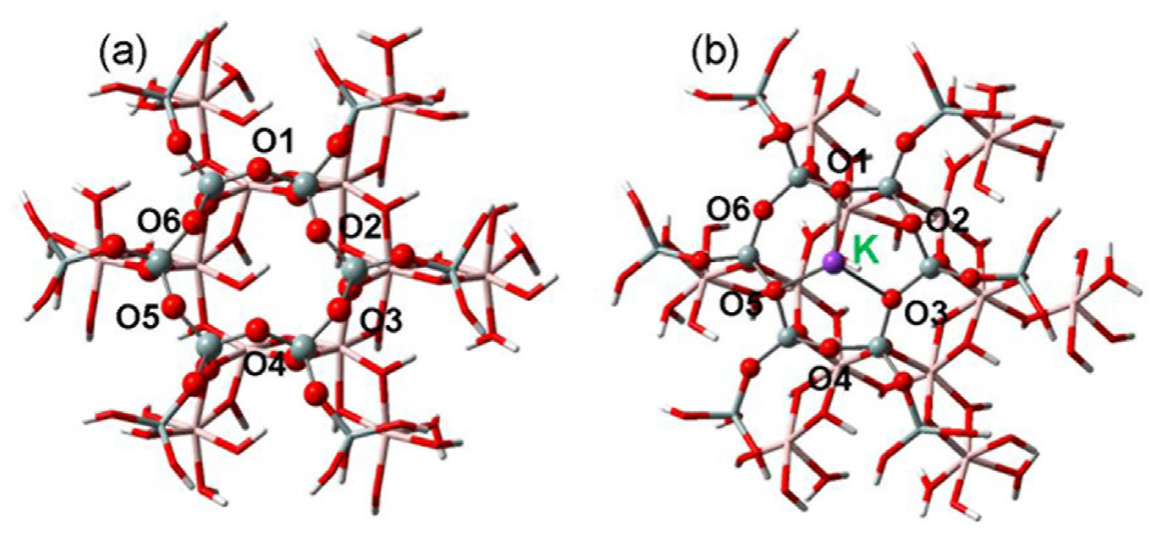
Cluster models for kaolinite mineral. (a) Neutral cluster model for the local structures of kaolinite mineral. (b) Charged cluster models for interacted structure with K^+^. The Si, O, H, Al and K atoms are displayed in cyan, red, white, rose pink and purple, respectively.

One of the most striking properties of NSCs is the carrying of abundant negative charges that is responsible for the exchange and adsorption of metal ions. For different aluminosilicate minerals, the tetrahedral SiO_4_ surfaces where metal ions are adsorbed [44] are rather close to each other, and hence cluster models with different negative charges were constructed from kaolinite, one of the most common minerals to us. The chemical formula of neutral kaolinite equals Al_2_Si_2_O_5_(OH)_4_. In this work, the kaolinite cluster models contain 12 Si and 12 Al atoms, and the boundary O atoms were saturated by H atoms, see Fig 1a. The excessive charges of kaolinite are mainly due to the deprotonation/protonation of the hydroxyl groups, which are dependent on the pH values of aqueous solutions [45–47]. In accord with the previous studies of aluminosilicates [48–51], the kaolinite cluster models were divided into two regions and simulated at different theoretical levels. The hexagonal ring of silica surface may interact directly with cations [44] and relating O and Si atoms were selected as the high-level region. As indicated in Fig 1a, the high-level region (represented as ball and stick) also includes the O atoms bonded to the hexagonal Si atoms as well as adsorbents, while the rest atoms of kaolinite cluster models were treated as the low-level region (in stick).

First-principles density functional calculations were performed with Gaussian09 software packages [52]. In agreement with our previous works [48, 49], the high- and low-level regions were described by the B3LYP/6-31+G(d,p) and B3LYP/3-21G methods, respectively [53,54]. On basis of optimized structures, NBO (natural bond orbital), electrostatic potential (ESP) and Hirshlfeld charge analyses were respectively made, and dipole moments were calculated by use of Hirshlfeld charges [55–57].

## Results and Discussion

### Aggregation kinetics and Hofmeister effects

NSCs are obviously more complex than montmorillonite previously studied [18]. X-ray diffraction patterns shown in S2 Fig indicate that NSC1 is composed of 2% quartz, 22% mica, 5% kaolinite, 48% illite, 23% vermiculite while NSC2 consists of 4% quartz, 13% mica, 15% illite, 24% montmorillonite, 34% vermiculite and 10% albite. All the constituents of NSC1 and NSC2 are negatively charged, which is also applicable for most NSCs. As a result, these two NSCs are preferential to interact with electrolyte cations rather than anions. Accordingly, their aggregation kinetics should be dominated by electrolyte cations, as has been confirmed before in the case of montmorillonite [18].

The hydrodynamic diameters of NSC1 in the various alkali ion solutions that increase with the experimental time are shown in S1 Fig, and on such basis, the total average aggregation (TAA) rates for NSCs can be calculated by [18],

$${\tilde{v}}_{T}(c_{0})=\frac{1}{t_{0}}\int_{0}^{t_{0}}\tilde{v}(t,c_{0})dt=\frac{1}{t_{0}}\int_{0}^{t_{0}}\frac{D(t)-D_{0}}{t}dt$$ (1)

where ${\tilde{v}}_{T}(c_{0})$ (nm/min) represents the TAA rate from *t* = 0 to an arbitrary time *t* (*t* > 0) and for a given time *t*_0_, it is equivalent to ${\tilde{v}}_{T}(c_{0})$. Note that *t*_0_ is selected with a given time interval during DLS measurements and its upper limit can be the ending time of the aggregation process. *c*_0_ (mmol/L) is the electrolyte concentration, *D*(*t*) (nm) is the hydrodynamic diameter of NSC aggregates at time *t* (*t* > 0), and *D*_0_ (nm) is the hydrodynamic diameter at the beginning (i.e. *t* = 0).

**Fig 2:**
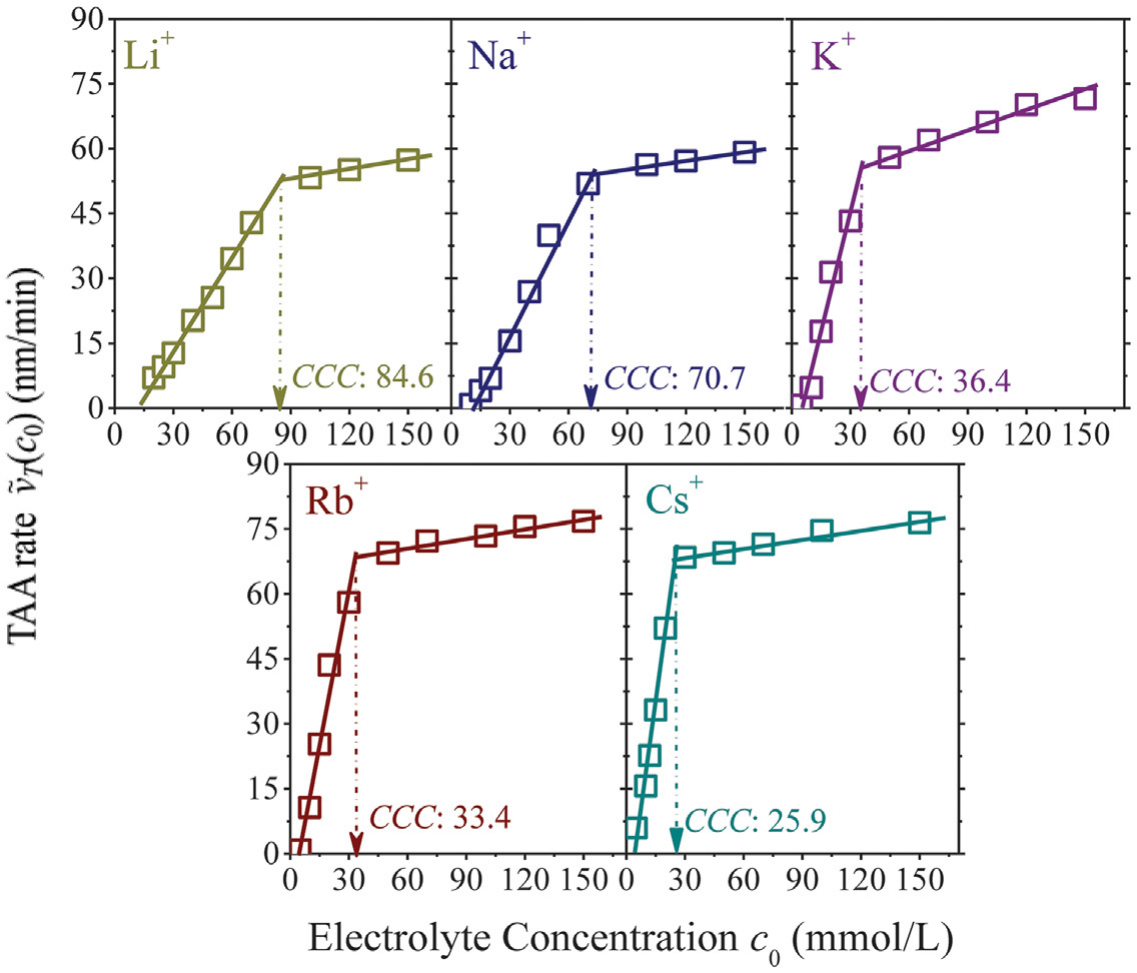
The total average aggregation (TAA) rate *ṽ*_*T*_ (*C*_0_) for NSC1 as function of the concentration *c*_0_ of alkali ion solutions.

Plots of the TAA rates vs. electrolyte concentrations are given in Fig 2, where strong Hofmeister effects have been detected for these five alkali ions. For instance, at 10 mmol/L, the TAA rates are equal to 0, 0.9, 4.8, 10.7 and 15.6 nm/min in Li^+^, Na^+^, K^+^, Rb^+^ and Cs^+^ solutions, respectively. Note that the aggregation process has not started in 10 mmol/L Li^+^ solutions. For each alkali ion solution, the TAA rates increase pronouncedly with electrolyte concentrations at first and then reach the plateau. Although much more complicated, NSC1 shows resembling aggregation kinetics as montmorillonite [18]. The TAA rates at low and high concentration regions are represented by two respective linear functions. Intersection for each plot is defined as *CCC* (critical coagulation concentration). The *CCC* values are equal to 84.6, 70.7, 36.4, 33.1 and 25.9 mmol/L for Li^+^, Na^+^, K^+^, Rb^+^ and Cs^+^, respectively. Thus, the Hofmeister effects during the aggregation of NSC1 should abide by the sequence of Cs^+^ > Rb^+^ > K^+^ >> Na^+^ >> Li^+^, in the same trend as that of montmorillonite [18].

The Hofmeister effects are further investigated by activation energies Δ*E*(*c*_0_), which have been correlated with the TAA rates through a combined use of reaction rate and Arrhenius expressions [18, 58],

$$\begin{array}{l} {\tilde{v}}_{T}(c_{0})=K\cdot c_{0}\cdot e^{-\frac{\Delta E(c_{0})}{RT}}(c_{0}\leq CCC)\\ {\tilde{v}}_{T}(c_{0})=K\cdot e^{-\frac{\Delta E(c_{0})}{RT}}(c_{0}\geq CCC) \end{array}$$ (2)

with

$$\begin{array}{l} K={\tilde{v}}_{T}(CCC)/CCC(c_{0}\leq CCC)\\ K={\tilde{v}}_{T}(CCC)(c_{0}\geq CCC) \end{array}$$ (3)

where *R* is the gas constant, and *T* is the absolute temperature. *K* can be regarded as a constant, whether the electrolyte concentration is below or above *CCC*.

The activation energies for the aggregation of NSC1 in the various alkali ion solutions are written as,

In Li^+^ solution:

$$\Delta E(c_{0})=-RT\text{ln}(-13.5/c_{0}+1.16)\begin{array}{ccc} & \left(c_{0}\leq 84.6\text{mmol/L}\right) & \end{array}$$ (4)

In Na^+^ solution:

$$\Delta E(c_{0})=-RT\text{ln}(-12.3/c_{0}+1.17)\begin{array}{ccc} & \left(c_{0}\leq 70.7\text{mmol/L}\right) & \end{array}$$ (5)

In K^+^ solution:

$$\Delta E(c_{0})=-RT\text{ln}(-6.20/c_{0}+1.17)\begin{array}{ccc} & \left(c_{0}\leq 36.4\text{mmol/L}\right) & \end{array}$$ (6)

In Rb^+^ solution:

$$\Delta E(c_{0})=-RT\text{ln}(-5.16/c_{0}+1.16)\begin{array}{ccc} & \left(c_{0}\leq 33.1\text{mmol/L}\right) & \end{array}$$ (7)

In Cs^+^ solution:

$$\Delta E(c_{0})=-RT\text{ln}(-4.89/c_{0}+1.19)\begin{array}{ccc} & \left(c_{0}\leq 25.9\text{mmol/L}\right) & \end{array}$$ (8)

**Fig 3:**
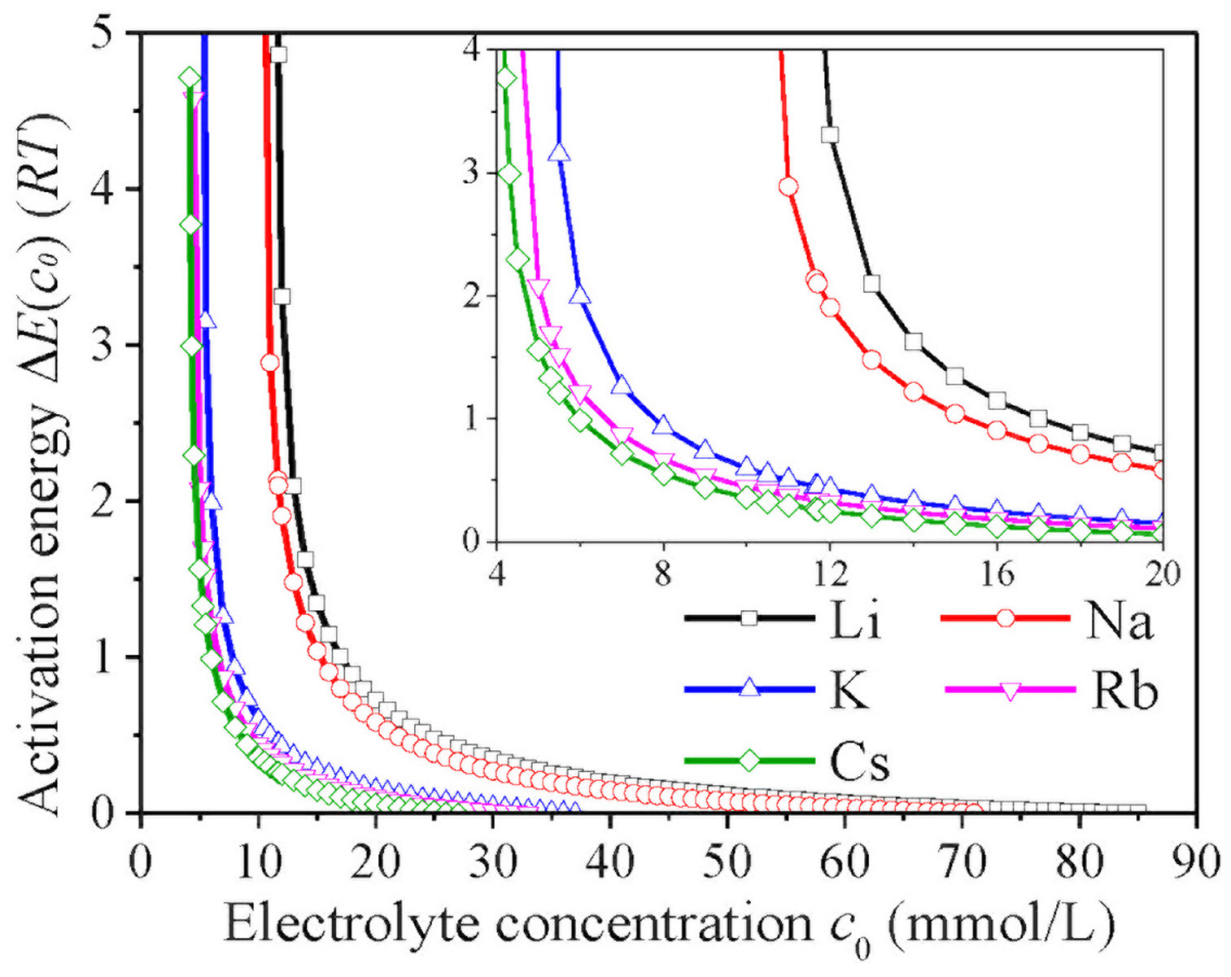
The activation energies Δ*E*(*c*_0_) for the aggregation of NSC1 as functions of electrolyte concentrations *c*_0_ for the various alkali ion solutions.

**Fig 4:**
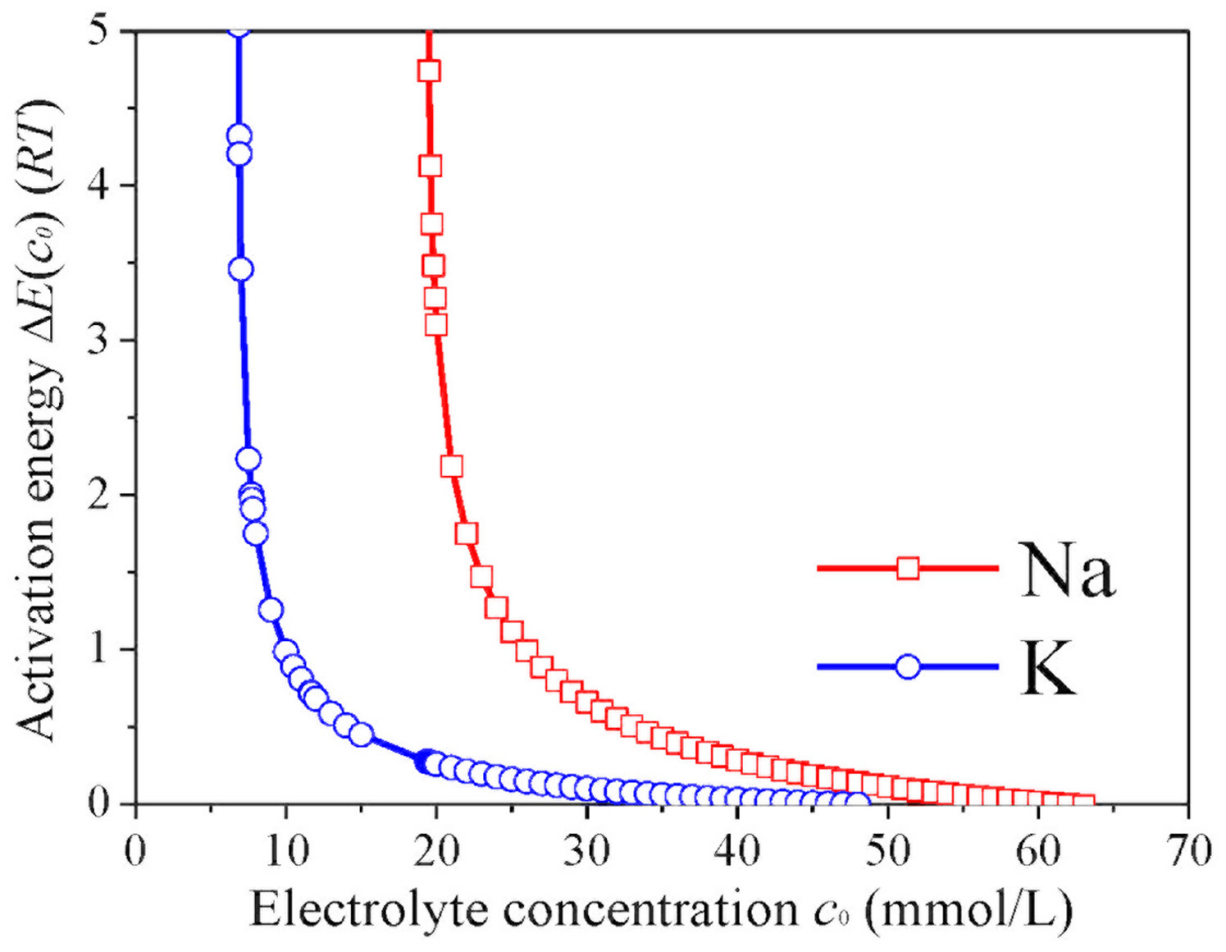
The activation energies Δ*E*(*c*_0_) for the aggregation of NSC2 as functions of electrolyte concentrations *c*_0_ for the various alkali ion solutions.

Eqs 4–8 describe the activation energies of five alkali ions below *CCC*. As indicated by the TAA rate plots (Fig 2) and Eq 2, the activation energies above *CCC* approach zero; that is, Δ*E*(*c*_0_) ≈ 0 for *c*_0_ ≥ *CCC*. For a given electrolyte concentration below *CCC*, the activation energies are significantly different for the various alkali ions and decrease as Li^+^ >> Na^+^ >> K^+^ > Rb^+^ > Cs^+^ (Fig 3), which is consistent with the results of montmorillonite [18]. The resembling kinetic behaviors between real and model colloids are further corroborated by the studies of NSC2 aggregation in NaNO_3_ and KNO_3_ solutions (S12 Fig). On basis of the hydrodynamic diameters, TAA rates and CCC values, Hofmeister effects resulting from NSC2 aggregation should be K^+^ > Na^+^. As indicated in Fig 4, at a given electrolyte concentration below *CCC*, the activation energies for Na^+^ are far larger than those for K^+^ and show good agreement with the results of NSC1 and montmorillonite.

Thus, for the aggregation of real and complex soil colloids (e.g., NSCs) in electrolyte solutions, Hofmeister series has been explicitly demonstrated to follow as Cs^+^ > Rb^+^ > K^+^ >> Na^+^ >> Li^+^. This is consistent with the sequences of ion exchange on montmorillonite [43] and montmorillonite aggregation [18]. Both ion exchange and aggregation processes are closely associated with the strength of ion adsorption. Generally, the negative charges of NSC surfaces are more screened by stronger ion adsorption, which further results in the lower activation energy for aggregation. In addition, Hofmeister effects can be evaluated quantitatively by activation energies, and their differences can be discerned for the various alkali ions; e.g., at 15 mmol/L, the activation energies for the aggregation of NSC1 are equal to 1.34*RT*, 1.04*RT*, 0.28*RT*, 0.21*RT* and 0.15*RT* for Li^+^, Na^+^, K^+^, Rb^+^ and Cs^+^, respectively.

### Mechanism for NSC aggregation

**Fig 5:**
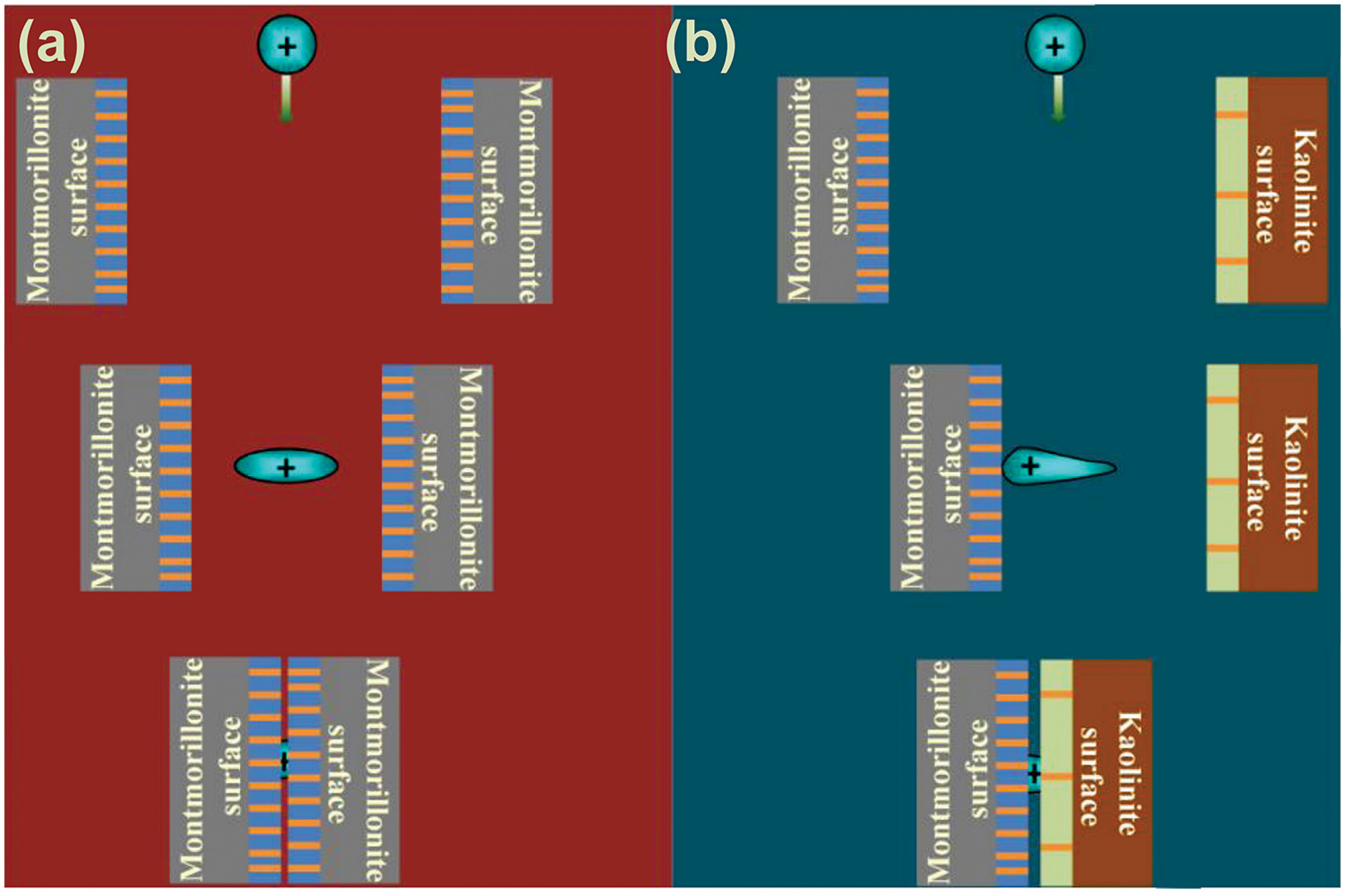
Schematic aggregation mechanisms for the aggregation of NSCs in electrolyte solutions. (a) The aggregation mechanisms of one-component NSC models. (b) The aggregation mechanisms of bi-component NSC models.

The mechanism for the aggregation of NSCs in alkali ion solutions is then demonstrated by first-principles density functional calculations. The cluster models of neutral mineral and its interaction with K^+^ are displayed in Fig 1. Different negative charges (-1 ∼ -5) are successively constructed in the mineral and adsorbed with K^+^, see the optimized structures in S13 Fig As indicated in S2 Table, the distances between K^+^ and adjacent mineral-O atoms first show some decline with negative charges (0 ∼ -4) while the further augment of negative charges (-4 ∼ -5) results in an opposite trend although slightly. Nonetheless, the interaction energies (*E*_int_) of K^+^ with minerals show a pronounced and yet monotonous increase with the negative charges; e.g., the *E*_int_ values are respectively calculated to be -400.2, -569.4, -742.4 and -1101.1 kJ/mol for 0, -1, -2 and -4 charges (Table 1). Hence, the aggregation kinetics of NSCs should be closely associated with the negative charges carried by minerals, and the aggregation mechanism should be as follows: mineral constituents with more negative charges are preferential to adsorb metal ions, and metal ions anchored this way then interact with colloid constituents with less negative charges. The aggregation processes will be cycled this way until finished. A bi-component NSC system (e.g., montmorillonite and kaolinite) is illustrated in Fig 5b, where montmorillonite instead of kaolinite has the priority to interact with alkali ions because of the substantially more abundance of negative charges [26]. This mechanism is significantly different from that of the mono-component colloid (e.g., montmorillonite). For the mono-component colloid, the negative charges are approximately equivalent and the interaction strengths of two colloidal particles with cations can be competitive. Accordingly, the aggregation mechanism proceeds as Fig 5a, where two colloidal particles approach cations almost at the same time. Apparently, NSCs are even much more complicated than the bi-component system of Fig 5b.

**Table 1:** Dipole moments of minerals (*μ*) with different charges, total interaction energies (*E*_int_) and electrostatic contributions (*E*_ele_) of K^+^ with minerals and dipole moments of the adsorbed K^+^ (*μ*_K_) based on Hirshlfeld population analyses.^*a*^

Minerals		K^+^/Minerals		
charge	*μ*	*E*_int_	*E*_ele_ (*E*_ele_′)^*b*^	*μ*_K_
0	60.16	-400.2	-0.294Π (-0.394Π)	0.55
-1	102.07	-569.4	-0.292Π (-0.389Π)	0.58
-2	192.43	-742.4	-0.289Π (-0.381Π)	0.62
-3	275.33	-938.2	-0.284Π (-0.371Π)	0.66
-4	338.99	-1101.1	-0.279Π (-0.361Π)	0.70
-5	424.89	-1283.4	-0.246Π (-0.316Π)	1.03

^*a*^ Units of dipole moments and interaction energies are Debye and kJ/mol, respectively;

^*b*^ *E*_ele_′ refers to electrostatic energy of K^+^ with only the six neighboring O atoms from minerals

As the aggregation mechanism is closely associated with the negative charges carried by minerals, electrostatic interactions are assumed to have played a significant role. The electrostatic interaction energies (*E*_ele_) of K^+^ with the adjacent SiO_4_ tetrahedra of minerals (relating O and Si atoms are marked in ball and stick, see Fig 1 and S13 Fig) are expressed as,

$$\Delta E_{ele}=\sum_{i}\frac{Q_{\text{i}}Q_{\text{K}}}{4\pi \varepsilon r}=\sum_{i}\frac{Q_{\text{i}}Q_{\text{K}}}{r}\Pi$$ (9)

where Q_i_ and Q_K_ refer to the Hirshlfeld charges of mineral O/Si atoms and K^+^, *r* is the distance of K^+^ with mineral O/Si atoms, and *ε* and Π represent the dielectric and Coulomb constants, respectively.

**Fig 6:**
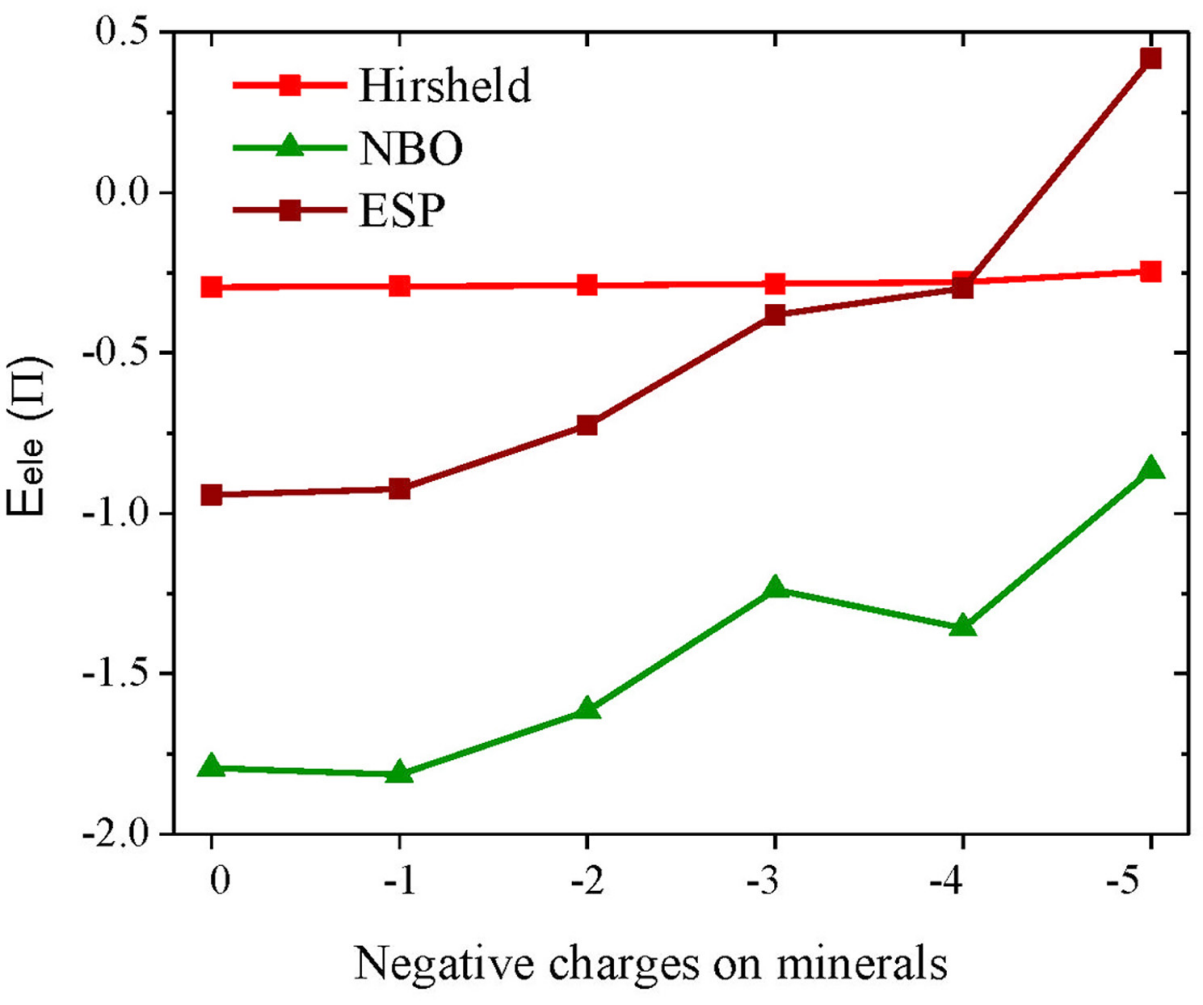
Electrostatic interaction energies (*E*_ele_) between K^+^ and minerals calculated by different atomic charge schemes.

Table 1 indicates that the electrostatic interaction energies (*E*_ele_ or *E*_ele_′) of K^+^ with minerals of different negative charges, which, to our surprise, do not increase but instead show a gradual decrease with the augment of negative charges of minerals. In addition, such a trend becomes even more apparent with more negative charges carried by minerals. Note that *E*_ele_′ represents the electrostatic interaction energies of K^+^ with only six adjacent O atoms of the hexagonal ring. Two other atomic charge schemes (NBO and ESP) are also used to calculate the electrostatic interaction energies (*E*_ele_), see Fig 6. Although the exact values differ significantly, the changing trends calculated by three charge schemes are consistent with each other. It explicitly shows that the electrostatic interaction energies (*E*_ele_) between minerals and metal ions generally decrease with the increase of negative charges carried by minerals. Accordingly, electrostatic interactions are unlikely to be the driving force for the aggregation of NSCs.

**Fig 7:**
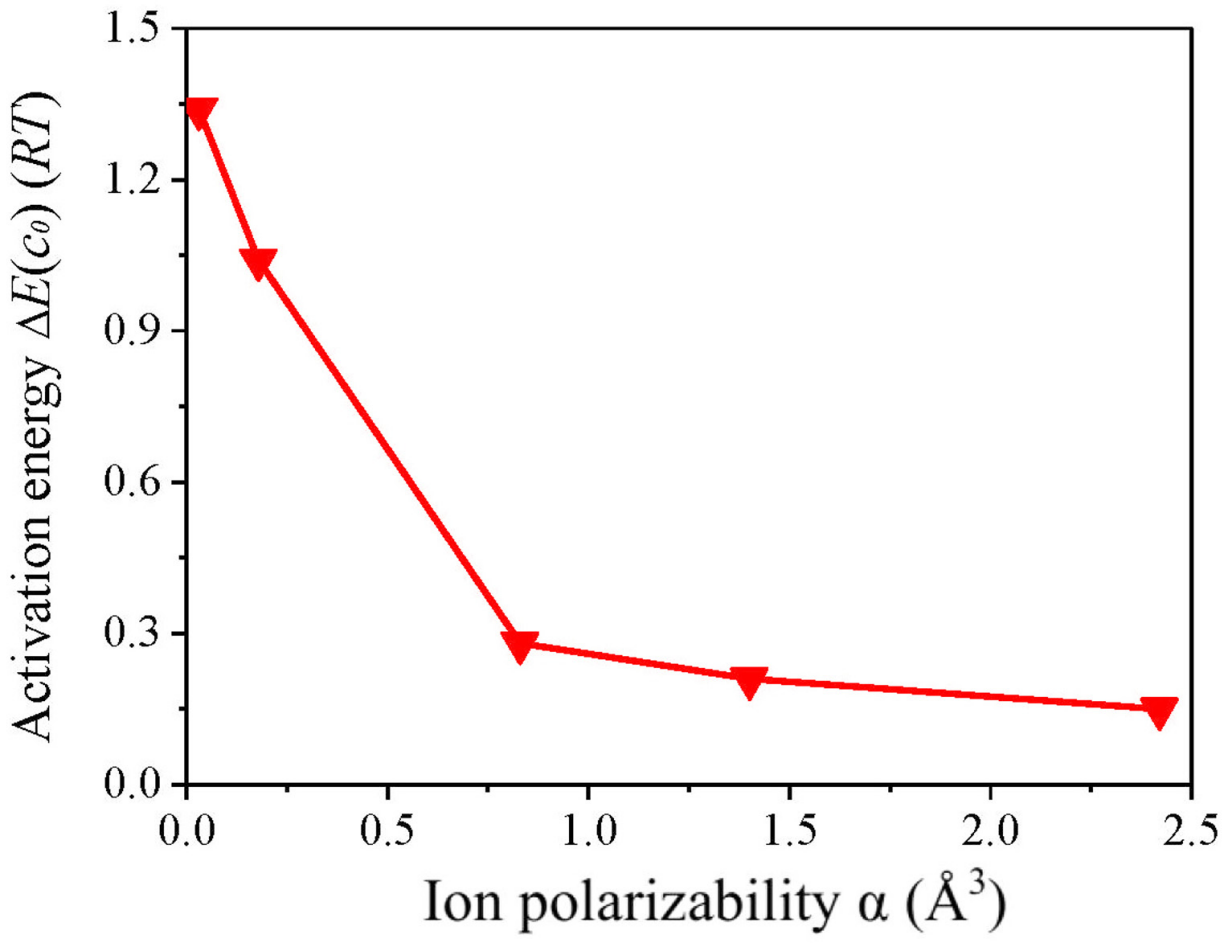
Correlation of the activation energies Δ*E*(*c*_0_ = 15 mmol/L) for the aggregation of NSC1 in alkali ion solutions and the polarizabilities α of alkali ions. The polarizability data have been taken from [59].

Polarization effect has been assumed to arouse Hofmeister effects for the aggregation of model colloid (montmorillonite), while no direct or convincing evidence has been given yet [18]. NSCs are composed of structurally distinct minerals and their aggregation processes are definitely more elusive. Owing to that all the constituents of NSC1 and NSC2 are negatively charged, their aggregation processes should be dominated by electrolyte cations as discussed earlier. Fig 7 presents that there is fine correlation between the activation energies and the polarizablities of alkali ions [56, 59]. Alkali ions with larger atomic nuclei are more significantly polarized by negatively charged minerals, consistent with the acquainted fact that alkali ions with larger atomic nuclei have less control for outer electrons. Stronger polarization effects to alkali ions substantially increase the interaction strengths with minerals and further facilitate their aggregation processes, which further result in the lower activation energies and stronger Hofmeister effects. This indicates that polarization effect should be the driving force for the aggregation of NSCs.

**Fig 8:**
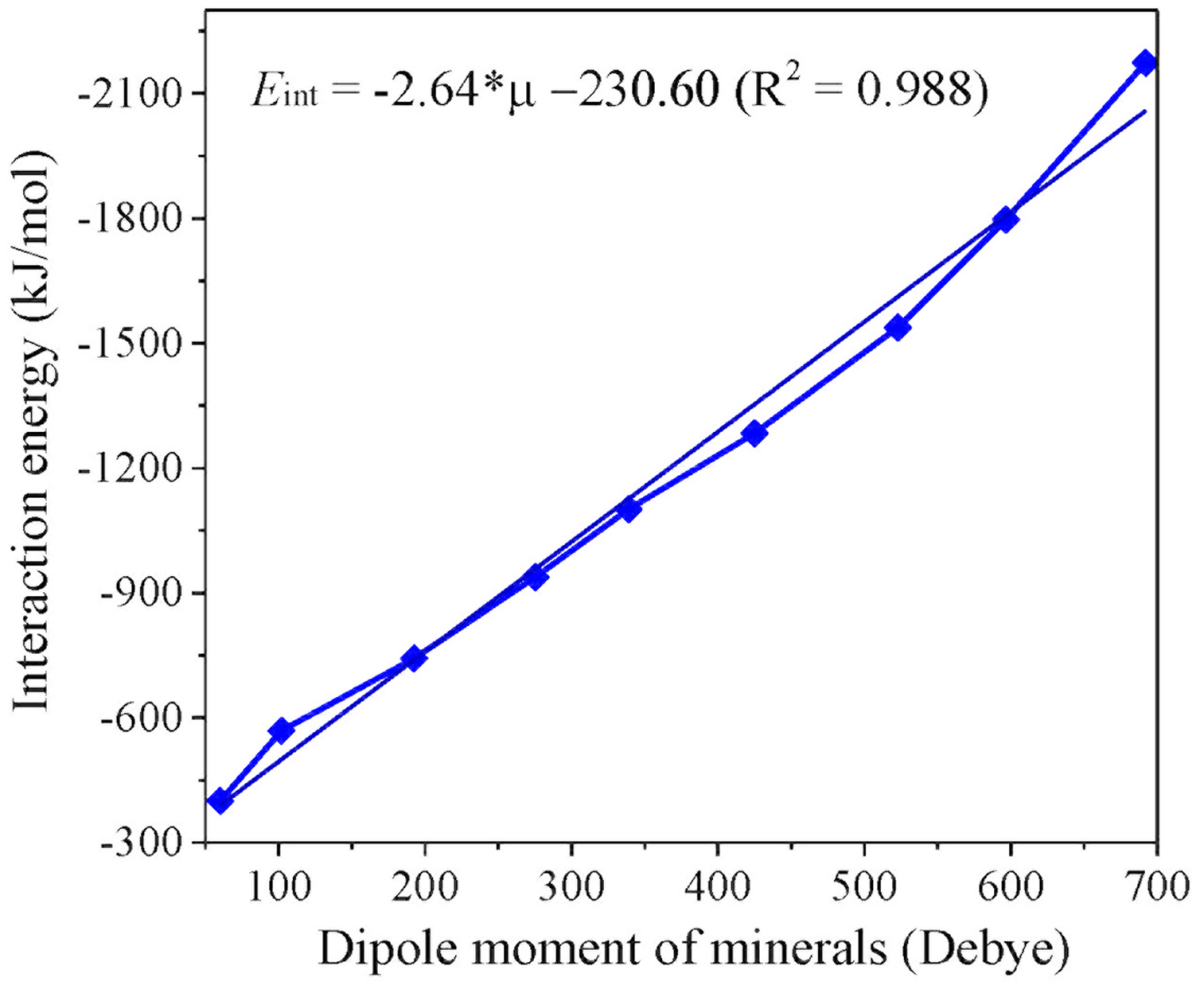
Correlation between the dipole moments (μ) of minerals with different charges and total interaction energies (*E*_int_) with K^+^.

As indicated in Table 1, the mineral cluster models with different negative charges have distinct dipole moments (μ). In addition, the μ values show a substantial and monotonous increase with the negative charges, which is in exactly the same trend with that of the interaction energies (*E*_int_) between metal ions and minerals (Fig 8). The further corroborates that polarization effect is the driving force for the aggregation of NSCs. Mineral constituents with more negative charges have larger polarization effects for adsorbents and correspond to stronger interaction strengths, which further result in their priority during the aggregation processes. As a result, polarization effect should be responsible for the strong Hofmeister effects arising during the aggregation processes, as further verified by the dipole moment calculations for adsorbed K^+^ ions (*μ*_K_). The *μ*_K_ values show a monotonous increase with the negative charges carried by mineral constituents (Table 1). The DLVO theory has recently experienced not a few failures owing to the neglect of non-DLVO forces [22–24, 60]. For charged particles, polarization effect is evidenced to be indispensable for explaining the experimental observations and can be the major reason for arousing the Hofmeister effects.

## Conclusions

In this work, we have presented the aggregation kinetics for complex colloids such as NSCs and quantitatively evaluated the Hofmeister effects implicated therein. Then the mechanism and driving force for the aggregation processes as well as origin of Hofmeister effects are demonstrated. The results obtained thus far can be extended to other charged systems that are ubiquitous in nature such as proteins, clays, colloids and membranes.

NSCs are obviously more complicated than montmorillonite but show resembling aggregation kinetics. In the case of NSCs, colloidal constituents with more negative charges are preferential to adsorb cations, and the significantly polarized cations then interact with those with less negative charges. The aggregation processes will be cycled this way until finished. It is quite different from the case of mono-component colloids, where particles should approach cations almost simultaneously. Although the aggregation processes are closely associated with the negative charges of colloids, electrostatic interactions, to our surprise, show a gradual decrease with the augment of negative charges and therefore play a minor role during the aggregation processes. Polarization effect has been clearly demonstrated to be the driving force for the aggregation of NSCs. On such basis, polarization effect is ascribed to be responsible for such strong Hofmeister effects during the aggregation of charged particles. This is further corroborated by the fine correlation between activation energies and polarizability data of the various alkali ions as well as the dipole moment calculations for minerals with different negative charges and for the alkali ions adsorbed on these charged minerals. Meanwhile, it indicates that why the prevailing DLVO theory is not applicable for colloidal systems because polarization effect is indispensable to describe relating Hofmeister effects. Thus, the present work represents a significant step towards the comprehension of Hofmeister effects that have been recognized as important as Mendel’s work was to genetics.

## Supporting Information

S1 Fig The time-evolution hydrodynamic diameters of NSC1 in LiNO_3_, NaNO_3_, KNO_3_, RbNO_3_, and CsNO_3_ solutions of different concentrations. The electrolyte concentrations from the bottom to the top are 20, 25, 30, 40, 50, 60, 70, 100, 120, and 150 mmol/L for Li^+^; 10, 15, 20, 30, 40, 50, 70, 100, 120, and 150 mmol/L for Na^+^; 5, 10, 15, 20, 30, 50, 70, 100, 120, and 150 mmol/L for K^+^; 5, 10, 15, 20, 30, 50, 70, 100, 120, and 150 mmol/L for Rb^+^; 5, 10, 12, 15, 20, 30, 50, 70, 100, and 150 mmol/L for Cs^+^, respectively.
(TIFF)

S2 Fig X-ray diffraction patterns for (a) NSC1 and (b) NSC2. (TIFF)

S3 Fig The time-evolution hydrodynamic diameters of NSC1 in LiNO_3_ solutions of different concentrations obtained from three independent DLS measurements. The electrolyte concentrations (mmol/L) are listed in the legends.
(TIFF)

S4 Fig The time-evolution hydrodynamic diameters of NSC1 in CsNO_3_ solutions of different concentrations obtained from three independent DLS measurements. The electrolyte concentrations (mmol/L) are listed in the legends.
(TIFF)

S5 Fig The TAA rates *ṽ*_*T*_ (*C*_0_) for NSC1 vs. the electrolyte concentrations *c*_0_ of LiNO_3_ solutions obtained from three independent DLS measurements. (TIFF)

S6 Fig The TAA rates *ṽ*_*T*_ (*C*_0_) for NSC1 vs. the electrolyte concentrations *c*_0_ of CsNO_3_ solutions obtained from three independent DLS measurements. (TIFF)

S7 Fig The TAA rates *ṽ*_*T*_ (*C*_0_) for NSC1 vs. the electrolyte concentrations *c*_0_ of LiNO_3_ solutions, where error bars are obtained for three independent DLS experiments. (TIFF)

S8 Fig The TAA rates *ṽ*_*T*_ (*C*_0_) for NSC1 vs. the electrolyte concentrations c_0_ of NaNO_3_ solutions, where error bars are obtained for three independent DLS experiments. (TIFF)

S9 Fig The TAA rates *ṽ*_*T*_ (*C*_0_) for NSC1 vs. the electrolyte concentrations c_0_ of KNO_3_ solutions, where error bars are obtained for three independent DLS experiments. (TIFF)

S10 Fig The TAA rates *ṽ*_*T*_ (*C*_0_) for NSC1 vs. the electrolyte concentrations c_0_ of RbNO_3_ solutions, where error bars are obtained for three independent DLS experiments. (TIFF)

S11 Fig The TAA rates *ṽ*_*T*_ (*C*_0_) for NSC1 vs. the electrolyte concentrations c_0_ of CsNO_3_ solutions, where error bars are obtained for three independent DLS experiments. (TIFF)

S12 Fig Time-evolution of the hydrodynamic diameters for the aggregation of the NSC2 in NaNO_3_ and KNO_3_ solutions. The electrolyte concentrations from the bottom to the top are 20, 30, 50, 60, 80, 100, and 120 mmol/L for Na^+^ and 10, 15, 20, 25, 30, 40, 50, 80, 100, and 120 mmol/L for K^+^, respectively.
(TIFF)

S13 Fig Structures of K^+^ interactions with kaolinite minerals of different charges. (a) -1; (b) -2; (c) -3; (d) -4; (e) -5.
(TIFF)

S1 Table The fitted equations of the TAA rates *ṽ*_*T*_ (*C*_0_) vs. the electrolyte concentrations c0 for the aggregation of NSC2 in LiNO_3_ and CsNO_3_ solutions respectively obtained from three independent DLS experiments. (DOCX)

S2 Table Distances between K^+^ and six adjacent O atoms from the hexagonal ring of kaolinite minerals with different charges. (DOCX)

S1 File Experimental details. S1.1. Preparation and characterization of NSCs; S1.2. X-ray diffraction experiments; S1.3. Reproducibility of experimental data; S1.4. The TAA rates and uncertainty bars.
(DOCX)

S2 File Hofmeister effects for the aggregation of NSC2. (DOCX)
