# Supplementary material for: Origin of Hofmeister Effects for Complex Systems
Source: PLoS One. 2015 Jul 22;10(7):e0128602. doi: 10.1371/journal.pone.0128602 (PMC4511582; doi:10.1371/journal.pone.0128602)
Supplement: S1 Table — (DOCX) [file pone.0128602.s014.docx]

**S1 Table. The fitted equations of the total average aggregation (TAA) rates *_T_*(*c*_0_) vs. electrolyte concentrations *c*_0_ for the aggregation of NSC1 in LiNO_3_ and CsNO_3_ solutions respectively obtained from three independent DLS measurements***^a^*

|  | *CCC* | *_T_*(*c*_0_) = (*c*_0_ ≤ *CCC*) | *_T_*(*c*_0_) = (*c*_0_ ≥ *CCC*) |
| --- | --- | --- | --- |
| LiNO_3_ | 84.6 | 0.7142*c*_0_-8.296 (R^2^ = 0.99) | 0.0807*c*_0_+45.29 (R^2^ = 1.00) |
|  | 83.1 | 0.7299*c*_0_-8.293 (R^2^ = 0.99) | 0.0582*c*_0_+46.59 (R^2^ = 1.00) |
|  | 83.8 | 0.7184*c*_0_-8.278 (R^2^ = 0.99) | 0.028*c*_0_+49.56 (R^2^ = 1.00) |
| CsNO_3_ | 25.6 | 3.128*c*_0_-12.88 (R^2^ = 0.98) | 0.0709*c*_0_+66.41 (R^2^ = 0.98) |
|  | 25.0 | 3.144*c*_0_-12.91 (R^2^ = 0.97) | 0.1064*c*_0_+63.09 (R^2^ = 0.99) |
|  | 25.3 | 3.128*c*_0_-12.41 (R^2^ = 0.98) | 0.0979*c*_0_+64.37 (R^2^ = 0.97) |

*^a^* Units of *_T_*(*c*_0_) and *CCC* are nm/min and mmol/L, respectively.
