# Supplementary material for: Origin of Hofmeister Effects for Complex Systems
Source: PLoS One. 2015 Jul 22;10(7):e0128602. doi: 10.1371/journal.pone.0128602 (PMC4511582; doi:10.1371/journal.pone.0128602)
Supplement: S2 Table — (DOCX) [file pone.0128602.s015.docx]

**S2 Table.** Distances between K^+^ and six adjacent O atoms from the hexagonal ring of kaolinite minerals with different charges*^a^*

| Charges of minerals | r(K-O1) | r(K-O2) | r(K-O3) | r(K-O4) | r(K-O5) | r(K-O6) | <r(K-O)>*^b^* |
| --- | --- | --- | --- | --- | --- | --- | --- |
| 0 | 2.836 | 3.097 | 2.702 | 3.133 | 2.710 | 3.529 | 3.001 |
| -1 | 2.760 | 2.982 | 2.673 | 3.170 | 2.733 | 3.493 | 2.968 |
| -2 | 2.751 | 2.944 | 2.626 | 3.116 | 2.713 | 3.477 | 2.938 |
| -3 | 2.722 | 2.906 | 2.603 | 3.091 | 2.690 | 3.419 | 2.905 |
| -4 | 2.714 | 2.916 | 2.610 | 3.057 | 2.660 | 3.333 | 2.882 |
| -5 | 2.742 | 2.943 | 2.618 | 3.035 | 2.659 | 3.345 | 2.890 |

*^a^* Unit of distances is Å;

*^b^* Average values for the indicated six K-O distances.
