# Supplementary material for: Origin of Hofmeister Effects for Complex Systems
Source: PLoS One. 2015 Jul 22;10(7):e0128602. doi: 10.1371/journal.pone.0128602 (PMC4511582; doi:10.1371/journal.pone.0128602)
Supplement: S1 File — S1.1. Preparation and characterization of NSCs; S1.2. X-ray diffraction experiments; S1.3. Reproducibility of experimental data; S1.4. The TAA rates and uncertainty bars. (DOCX) [file pone.0128602.s016.docx]

**S1. Experimental details**

**S1.1. Preparation and characterization of NSCs**

The combined method proposed by Li et al.^1^ was used and the surface charge densities for NSC1 and NSC2 were determined to be 0.289 and 0.353 C/m^2^, respectively. NSCs were prepared according to the following procedures. Firstly, the NSC particles were air-dried and screened through the 0.25 mm sieve, and 50 grams were transferred to a 500 mL beaker. Secondly, after addition of 500 mL ultrapure water, the NSCs were adjusted to pH = 7.5 with 500 mmol/L KOH and dispersed for 15 min using a probe-type ultrasonic homogenizer (Scientz-IID, Ningbo, China). Then, the dispersed NSCs were transferred into a 5 L beaker and diluted to 5 L with ultrapure water. After being laid for 33 days (298 K), the < 200 nm NSCs were collected using the static sedimentation method^2^, and their concentrations were measured by the oven drying method to be 0.88 and 0.84 g/L, respectively. Finally, the pH values were adjusted to 8.0 by 10 mmol/L KOH and then the NSCs were diluted 10 times that got ready for dynamic light scattering (DLS) measurements (S1 Fig.).

**S1.2. X-ray diffraction experiments**

The compositions of NSC1 and NSC2 were estimated in the air-dried state, with use of the X-ray diffraction technique (XD-3, Beijing Purkinje General Instrument Co., Ltd., Beijing, China). The Ni-filtered Cu Kα radiation (36 kV, 20 mA) was used, and a range of 2^o^ to 65^o^ was scanned at a rate of 2^o^/min. The X-ray diffraction patterns were collected in S2 Fig..

**S1.3. Reproducibility of experimental data**

The DLS experiments for the aggregation of NSCs in alkali ion solutions have been repeated two times. As indicated in S3 and S4 Figs., the electrolyte concentrations used in the three independent groups are not exactly the same, and this corroborates the applicability of the obtained results to the whole concentration ranges. Here we choose the aggregation of NSC1 in Li^+^ and Cs^+^ solutions to account for the reproducibility of experimental data. Based on the results of hydrodynamic diameter growths (S3 and S4 Figs.), the total average aggregation rates (TAA rate, *_T_*(*c*_0_)) are calculated against the electrolyte concentrations (*c*_0_) and then shown in S5 and S6 Figs.. S1 Table lists the fitted equations of *_T_*(*c*_0_) vs. *c*_0_, and it has been found that for a given alkali ion, the linear functions respectively obtained by the three independent groups quite resemble each other, and the slopes of their reaction limited cluster aggregation (RLCA) regimes (*c*_0_ ≤ *CCC*) deviate less than 0.02. Besides, the *CCC* values of the three independent groups are very close to each other, equaling 84.6, 83.1, and 83.8 mmol/L for Li^+^ and 25.6, 25.3, and 25.0 mmol/L for Cs^+^, respectively. All these results indicate that the data reported in the text are accurate and have a good reproducibility.

**S1.4. The total average aggregation (TAA) rates and uncertainty bars**

Based on the data of hydrodynamic diameter growths given in S1 Fig., the total average aggregation (TAA) rates *_T_*(*c*_0_) of NSC1 in Li^+^, Na^+^, K^+^, Rb^+^ and Cs^+^ solutions have been calculated against the electrolyte concentrations, see Fig. 1. As indicated above (S1.3. Reproducibility of experimental data), the DLS experiments have been repeated two times. The TAA rates of each DLS experiment can be obtained as a function of electrolyte concentrations (S5 and S6 Figs.), and their values are averaged and then shown in S7 ~ S11 Figs.. For the other two DLS measurements, the electrolyte concentrations may occasionally be different from those reported in the text, and in such cases, the TAA rates obtained from the fitted equations are used instead. Note that from S1 Table, it can be seen that the linear relationship between the TAA rates vs. electrolyte concentrations is quite accurate for each DLS experiment, with the coefficient of determination (R^2^) approaching 1.00.

The error bars representing the standard deviations of the TAA rates of three independent DLS experiments are also calculated and shown in S7 ~ S11 Figs..

**References:**

1. H. Li, J. Hou, X. M. Liu, R. Li, H. L. Zhu, and L. S. Wu, *Soil Sci*. *Soc*. *Am*. *J*., 2011, **75**, 2128-2135.

2. Y. Xiong, J. F. Chen, and J. S. Zhang, *Soil colloid (Book 2): Methods for soil colloid research*, Science Press: Beijing, 1990.
