# Supplementary material for: Origin of Hofmeister Effects for Complex Systems
Source: PLoS One. 2015 Jul 22;10(7):e0128602. doi: 10.1371/journal.pone.0128602 (PMC4511582; doi:10.1371/journal.pone.0128602)
Supplement: S2 File — (DOCX) [file pone.0128602.s017.docx]

**S2. Hofmeister effects for the aggregation of NSC2**

To corroborate the resemblance between real and model systems, the aggregation kinetics of NSC2 have also been studied. As described in the text, NSC2 consist of ca. 4% quartz, 13% mica, 15% illite, 24% montmorillonite, 34% vermiculite and 10% albite. S13 Fig. shows that the time-dependent hydrodynamic diameters for the aggregation of NSC2 in NaNO_3_ and KNO_3_ solutions. The TAA rates and *CCC* values for the aggregation of NSC2 are calculated in the same way as those of NSC1. The *CCC* values for Na^+^ and K^+^ are equal to 47.4 and 61.9 mmol/L, respectively. Accordingly, Hofmeister effects have also been detected for the aggregation of NSC2, and its order based on the hydrodynamic diameters, TAA rates and CCC values should be K^+^ > Na^+^, consistent with the results of NSC1.

By means of eq. (2), the activation energies for the aggregation of NSC2 in Na^+^ and K^+^ solutions are obtained,

In Na^+^ solution:

$$\Delta E(c_{0})=-RT\ln(-28.3/c_{0}+1.46)\begin{matrix} & (c_{0}\leq61.9\text{mmol/L}) & \end{matrix}(\text{S}1)$$

In K^+^ solution:

$$\Delta E(c_{0})=-RT\ln(-7.97/c_{0}+1.17)\begin{matrix} & (c_{0}\leq47.4\text{mmol/L}) & \end{matrix}(\text{S}2)$$

At above *CCC*, the activation energies for the aggregation of NSC2 are nearly zero (i.e., Δ*E*(*c*_0_) ≈ 0 for *c*_0_ ≥ *CCC*) because of the TAA rates for *c*_0_ ≥ *CCC* are nearly constant, which quite resemble the scenarios of NSC1 and montmorillonite colloids. It can be seen from Fig. 4 that at any electrolyte concentration below *CCC*, the activation energies are apparently different in Na^+^ and K^+^ solutions and this clearly indicates the presence of Hofmeister effects for the aggregation of NSC2. The activation energies for Na^+^ are far larger than those for K^+^ (i.e., Na^+^ >> K^+^), in good agreement with the results of NSC1. Similarly, the activation energies that reflect Hofmeister effects can be determined at any given electrolyte concentration; e.g., at 20 mmol/L equal to 3.10*RT* and 0.26*RT* for Na^+^ and K^+^, respectively. These substantialize that the Hofmeister effects for the aggregation of NSCs in electrolyte solutions can be qualitatively and quantitatively described by use of activation energies; meanwhile, “huge gaps” between model and real systems can be filled by use of the descriptor − activation energy.
